# Burden of Modifiable Risk Factors in Young-Onset Cryptogenic Ischemic Stroke by High-Risk Patent Foramen Ovale

**DOI:** 10.1161/STROKEAHA.124.049855

**Published:** 2025-04-17

**Authors:** Jukka Putaala, Nicolas Martinez-Majander, Michelle Leppert, Lauri Tulkki, Jani Pirinen, Heli Tolppanen, Tomi Sarkanen, Marko Virtanen, Nina Jaakonmäki, Pekka Jäkälä, Marja Hedman, Petra Redfors, Odd Bech-Hanssen, Ulla Junttola, Juha Huhtakangas, Pauli Ylikotila, Riikka Lautamäki, Ulf Schminke, Bettina von Sarnowski, Raila Busch, Nilüfer Yesilot, Mine Sezgin, Ulrike Waje-Andreassen, Sahrai Saeed, Ana Catarina Fonseca, André Paula, Laura Amaya Pascasio, Patricia Martínez-Sánchez, Janika Kõrv, Piibe Muda, Phillip Ferdinand, Cheryl Oxley, Diana Zakarkaitė, Kristina Ryliškienė, Alessandro Pezzini, Carlo Mario Lombardi, Radim Líčeník, Marialuisa Zedde, Teresa Grimaldi, Georgios Tsivgoulis, Juha Sinisalo, Eva Gerdts, Turgut Tatlisumak

**Affiliations:** Department of Neurology (J. Putaala, N.M.-M., L.T.), Helsinki University Hospital and University of Helsinki, Finland.; Department of Cardiology, Heart and Lung Center (J. Pirinen, H.T., J.S.), Helsinki University Hospital and University of Helsinki, Finland.; Department of Neurology, University of Colorado School of Medicine, Aurora (M.L.).; Department of Neurology, Tampere University Hospital, Wellbeing Services County of Pirkanmaa, and Faculty of Medicine and Health Technology, Tampere University, Finland (T.S.).; Heart Hospital, Tampere University Hospital, Finland (M.V.).; Neurocenter Neurology (N.J., P.J.), Kuopio University Hospital, Finland.; Heart Center (M.H.), Kuopio University Hospital, Finland.; University of Eastern Finland (N.J., P.J.).; Department of Neurology, Sahlgrenska University Hospital and Department of Clinical Neuroscience, Institute of Neuroscience and Physiology (P.R., T.T.), Sahlgrenska Academy at University of Gothenburg, Sweden.; Department of Clinical Physiology, Institute of Medicine (O.B.-H.), Sahlgrenska Academy at University of Gothenburg, Sweden.; Department of Neurology, Oulu University Hospital and University of Oulu, Finland (U.J., J.H.).; Department of Neurology, Turku University Hospital and University of Turku, Finland (P.Y.).; Heart Center, Turku University Hospital, Finland (R. Lautamäki).; Department of Neurology (U.S., B.v.S.), University Medicine Greifswald, Germany.; Department of Internal Medicine B (Cardiology) (R.B.), University Medicine Greifswald, Germany.; Department of Neurology (N.Y.), Istanbul University Faculty of Medicine, Turkey.; Department of Cardiology (M.S.), Istanbul University Faculty of Medicine, Turkey.; Department of Neurology (U.W.-A.), Haukeland University Hospital, Bergen, Norway.; Department of Heart Disease (S.S.), Haukeland University Hospital, Bergen, Norway.; Hospital Santa Maria, Faculty of Medicine, University of Lisbon, Portugal (A.C.F., A. Paula).; Department of Neurology, Torrecardenas University Hospital, University of Almería, Spain (L.A.P., P.M.-S.).; Department of Neurology and Neurosurgery (J.K.), University of Tartu, Estonia.; Department of Cardiology (P.M.), University of Tartu, Estonia.; Neurosciences (P.F.), University Hospitals of North Midlands NHS Trust, Stoke-on-Trent, United Kingdom.; Cardiac Physiology Services (C.O.), University Hospitals of North Midlands NHS Trust, Stoke-on-Trent, United Kingdom.; Faculty of Medicine, Center of Neurology, Vilnius University, Lithuania (D.Z., K.R.).; Department of Medicine and Surgery, University of Parma and Stroke Care Program, Department of Emergency, Parma University Hospital, Italy (A. Pezzini).; Cardiology, ASST Spedali Civili Brescia and Department of Medical and Surgical Specialties, Radiological Sciences, and Public Health, University of Brescia, Italy (C.M.L.).; North West Anglia NHS Foundation Trust, Acute Stroke Centre, United Kingdom (R. Licenik).; Neurology Unit, Stroke Unit (M.Z.), Azienda Unità Sanitaria Locale-IRCCS Reggio Emilia, Italy.; Cardiology Unit (T.G.), Azienda Unità Sanitaria Locale-IRCCS Reggio Emilia, Italy.; Second Department of Neurology, National & Kapodistrian University of Athens, “Attikon” University Hospital, Greece (G.T.).; Department of Clinical Science, Center for Research on Cardiac Disease in Women, University of Bergen, Norway (E.G.).; Tartu University Hospital, Tartu, Estonia; Tartu University Hospital, Tartu, Estonia; Tartu University Hospital, Tartu, Estonia; Helsinki University Hospital, Helsinki, Finland; Helsinki University Hospital, Helsinki, Finland; Helsinki University Hospital, Helsinki, Finland; Helsinki University Hospital, Helsinki, Finland; Helsinki University Hospital, Helsinki, Finland; Helsinki University Hospital, Helsinki, Finland; Helsinki University Hospital, Helsinki, Finland; Kuopio University Hospital and University of Eastern Finland, Kuopio, Finland; Kuopio University Hospital and University of Eastern Finland, Kuopio, Finland; Kuopio University Hospital and University of Eastern Finland, Kuopio, Finland; Tampere University Hospital, Tampere, Finland; Tampere University Hospital, Tampere, Finland; Turku University Hospital and University of Turku, Turku, Finland; Oulu University Hospital, Oulu, Finland; Oulu University Hospital, Oulu, Finland; Oulu University Hospital, Oulu, Finland; Attikon University Hospital, Athens, Greece; Attikon University Hospital, Athens, Greece; Azienda Unità Sanitaria Locale–IRCCS, Reggio Emilia, Italy; University of Brescia, Brescia, Italy; University of Brescia, Brescia, Italy; Vilnius University Hospital, Vilnius, Lithuania; Vilnius University Hospital, Vilnius, Lithuania; Vilnius University Hospital, Vilnius, Lithuania; Radboud University Medical Center, Nijmegen, the Netherlands; Radboud University Medical Center, Nijmegen, the Netherlands; Radboud University Medical Center, Nijmegen, the Netherlands; Radboud University Medical Center, Nijmegen, the Netherlands; Haukeland University Hospital, Bergen, Norway; Hospital de Santa Maria, University of Lisbon, Lisbon, Portugal; Hospital de Santa Maria, University of Lisbon, Lisbon, Portugal; Hospital de Santa Maria, University of Lisbon, Lisbon, Portugal; Torrecardenas University Hospital, Spain; Torrecardenas University Hospital, Spain; Torrecardenas University Hospital, Spain; Sahlgrenska Academy at University of Gothenburg and Sahlgrenska University Hospital, Gothenburg, Sweden; Sahlgrenska Academy at University of Gothenburg and Sahlgrenska University Hospital, Gothenburg, Sweden; Sahlgrenska Academy at University of Gothenburg and Sahlgrenska University Hospital, Gothenburg, Sweden; Sahlgrenska Academy at University of Gothenburg and Sahlgrenska University Hospital, Gothenburg, Sweden; Sahlgrenska Academy at University of Gothenburg and Sahlgrenska University Hospital, Gothenburg, Sweden; Sahlgrenska Academy at University of Gothenburg and Sahlgrenska University Hospital, Gothenburg, Sweden; Istanbul University, Istanbul, Turkey; Royal Stoke University Hospital, Stoke-on-Trent, United Kingdom; Royal Stoke University Hospital, Stoke-on-Trent, United Kingdom; Peterborough City Hospital, Peterborough, United Kingdom; Peterborough City Hospital, Peterborough, United Kingdom; Peterborough City Hospital, Peterborough, United Kingdom; Peterborough City Hospital, Peterborough, United Kingdom

**Keywords:** foramen ovale, patent, heart septal defects, ischemic stroke, migraine with aura, risk factors

## Abstract

**BACKGROUND:**

The incidence of young-onset ischemic stroke is rising, driven by cryptogenic ischemic stroke (CIS) and patients without vascular risk factors. This study examines the burden and associations of modifiable traditional, nontraditional, and female sex–specific risk factors with young-onset CIS, stratified by clinically relevant patent foramen ovale (PFO), defined by high-risk features of atrial septal aneurysm or large right-to-left shunt.

**METHODS:**

We enrolled consecutive patients aged 18 to 49 years with recent CIS and frequency-matched stroke-free controls of the same age and sex from 19 European sites. Logistic regression assessed the association of risk factor counts (12 traditional, 10 nontraditional, 5 female sex–specific) and individual risk factors, stratified by PFO. Analyses were stratified by sex and age (18–39 and 40–49 years), with computation of population-attributable risk.

**RESULTS:**

We included 523 patients (median age, 41 years; 47.3% women; 196 [37.5%] with PFO) and 523 controls. In patients with CIS without PFO, each additional traditional (odds ratio, 1.417 [95% CI, 1.282–1.568]), nontraditional (odds ratio, 1.702 [95% CI, 1.338–2.164]), and female sex–specific risk factor (odds ratio, 1.700 [95% CI, 1.107.1–2.611]) increased CIS risk. For patients with CIS with PFO, each traditional risk factor increased the risk (odds ratio, 1.185 [1.057–1.328]), but only nontraditional risk factors remained significant when fully adjusted (odds ratio, 2.656 [2.036–3.464]). Population-attributable risks for CIS without PFO were 64.7%, 26.5%, and 18.9% for traditional, nontraditional, and female sex–specific risk factors. For CIS with PFO, population-attributable risks were 33.8%, 49.4%, and 21.8%, respectively. Migraine with aura was the most significant contributor, with population-attributable risks of 45.8% for CIS with PFO and 22.7% for CIS without PFO, showing a stronger impact in women.

**CONCLUSIONS:**

Despite the initial cryptogenic label of these strokes, traditional risk factors significantly contribute to CIS without PFO, while nontraditional factors seem more critical for CIS with PFO. Migraine with aura plays a prominent role in young-onset CIS development, particularly in women.

**REGISTRATION:**

URL: https://www.clinicaltrials.gov; Unique identifier: NCT01934725.

Ischemic stroke incidence among younger adults has been rising over the last few decades.^1^ This increase may be partly attributed to a concomitant rise in the prevalence of known vascular risk factors.^2^ However, recent research suggests that nontraditional risk factors could be more significant drivers for this change. This hypothesis is supported by concurrently declining rates of myocardial infarction and sudden cardiac death among young adults, which are also strongly associated with traditional risk factors, and the observation that the proportion of cryptogenic ischemic strokes (CIS) has increased.^3^ Prior large case-control studies assessing risk factors for young-onset ischemic stroke have predominantly focused on traditional risk factors, demonstrating strong associations with these factors.^4–12^ However, recent research suggests that nontraditional risk factors may have a more profound impact on the increasing incidence of ischemic strokes, especially among younger individuals and women.^13^ Notably, the multiplicative effect of several risk factors combined—for example, a combination of behavioral risk factors including low physical activity, alcohol intake, and smoking—may be more significant than individual risk factors, as highlighted in a few studies focusing on young-onset strokes.^8,13,14^

Limited data from studies stratifying by stroke pathogenesis or focusing on CIS suggest associations of several individual risk factors—such as hypertension, diabetes, current smoking, heavy alcohol consumption, abdominal obesity, low physical activity, and migraine with aura—with young-onset CIS.^8,9,15–17^ However, data on the combined influence of traditional, nontraditional, and female sex–specific risk factors in the context of young-onset CIS are lacking. An important consideration when assessing phenotypes and potential causes of young stroke patients is the presence of a patent foramen ovale (PFO). Yet, previous large case-control studies of young-onset strokes have struggled to consistently classify PFO-associated strokes due to the uncertainty in attributing the cause. Consequently, the majority were labeled traditionally as cryptogenic, with a minority as cardioembolic. As a result, the potential interactions with risk factors have not been adequately addressed when stratifying by the presence of a PFO. It is known that young patients with CIS and PFO are less likely to have traditional risk factors and more likely to have nontraditional risk factors, especially migraine.^18,19^ Furthermore, recent research demonstrates that specific high-risk features of PFO—particularly atrial septal aneurysm or a large-sized shunt—should be considered in assessing the causal role of PFO and classification of this phenotypic trait.^20^

To our knowledge, no studies thus far have assessed the influences of a comprehensive set of risk factors on young-onset CIS, in the context of a clinically relevant PFO, defined as those with high-risk features. In this international case-control study, we evaluated the burden and strength of association of traditional, nontraditional, and female sex–specific risk factors in young-onset CIS, stratified by the presence or absence of PFO, as well as by sex and age groups.

## Methods

In this case-control study, we included consecutive young patients aged 18 to 49 years with CIS from the SECRETO study (Searching for Explanations for Cryptogenic Stroke in the Young: Revealing the Triggers, Causes, and Outcome) and an equal number of age- and sex-matched stroke-free controls. Participants were enrolled across 19 European centers between November 2013 and January 2022.^21^ Study sites were instructed to enroll consecutive patients among all acute stroke patients presenting to the hospital during the active recruitment phase. Ethical approval was obtained from local committees, and written informed consent was secured from all participants. This article follows the STROBE (Strengthening the Reporting of Observational Studies in Epidemiology) reporting guideline (www.strobe-statement.org). Data supporting the findings of this study are available from the corresponding author upon reasonable request.

Patients underwent standardized and timely etiologic examinations and were eligible (labeled as cryptogenic stroke) when they had A-S-C-O (atherosclerosis, small vessel disease, cardiac source, other cause) classification as the absence of disease (grade 0), or any of grade II (causality uncertain) or grade III (unlikely a direct cause) pathology applying the diagnostic testing of the highest level of evidence.^22^ All patients with PFO were included throughout the study period as data on the factors increasing the causality of PFO accrued over the years of enrollment and to ensure enrollment of the entire spectrum of patients with CIS with PFO. Etiologic investigations included brain magnetic resonance imaging, imaging of intracranial and extracranial vessels with either computed tomography angiography or magnetic resonance angiography, routine laboratory testing, 12-lead ECG, and continuous ECG for at least 24 hours. Transthoracic and transesophageal echocardiography studies were performed according to a standardized protocol.^23^ Ancillary etiologic testing in patients was carried out at the discretion of the attending physician.

PFO was diagnosed with color Doppler imaging showing spontaneous right-to-left shunt or the shift of shunt direction during Valsalva maneuver. PFO confirmation required a bubble study with microbubbles visualized in the left atrium during the first 3 to 5 cardiac cycles in transesophageal echocardiography. Selected sites also used additional transcranial Doppler bubble screens according to consensus guidelines to detect and quantify right-to-left shunt.^24^ Transcranial Doppler bubble screen was performed both at rest and with the Valsalva maneuver unless a severe right-to-left shunt was present at rest. A clinically relevant PFO was defined as a PFO with high-risk features including an atrial septal aneurysm or a large-sized shunt (≥25 microbubbles crossing the atrial septum in transesophageal echocardiography or detected in transcranial Doppler bubble screen).

Stroke-free control subjects, frequency-matched for sex and age (±5 years), from the same region, was identified locally at each study center. Due to differing legislation across study sites, sources for identifying control subjects were not standardized. They included random searches through population registers where feasible, and community controls including patients’ unrelated proxies and hospital staff unrelated to the study. None of the control subjects was hospitalized. The absence of a prior stroke was verified using the Questionnaire for Verifying Stroke-Free Status^25^ and a review of medical records. All stroke-free controls attended a study visit including interviews, anthropometric measurements, and review of medical records.

Clinical history was gathered from all patents and controls through medical records and structured interviews. Low level of education was classified as either primary or lower secondary education, or upper secondary education. As traditional risk factors, we considered those documented in the INTERSTROKE study (Global and Regional Effects of Potentially Modifiable Risk Factors Associated With Acute Stroke in 32 Countries)^11^ and focused the analysis on modifiable or potentially modifiable risk factors. Hypertension was defined as a prior diagnosis, antihypertensive medication use, or a mean of 2 office blood pressure measures ≥140/90 at the study visit. Diabetes was defined as a prior diagnosis or antidiabetic medication. Hypercholesterolemia was defined as a prior diagnosis of hypercholesterolemia or lipid-lowering medication. Current smoking was defined as smoking at least 1 cigarette per day on average. Cardiovascular disease was defined as a history of coronary heart disease, congestive heart failure, peripheral arterial disease, valvular or aortic disease, and obstructive sleep apnea as a prior diagnosis. Abdominal obesity was defined as a waist-to-hip ratio as >0.85 in women and >0.90 in men. An unhealthy diet was defined as a score of <25 on the modified version of the Mediterranean Diet Score.^26^ Physical inactivity was assessed using the short version of the International Physical Activity Questionnaire,^27^ defined as <1500 metabolic equivalents per week. Alcohol consumption was assessed using an adaptation of the World Health Organization Alcohol, Smoking and Substance Involvement Screening Test.^28^ Heavy alcohol consumption was defined as >7 units per week for women and >14 units per week for men, or binge drinking (≥5 units per instance for women and ≥7 units per instance for men) at least twice a month,^17^ according to Substance Abuse and Mental Health Services Administration, US Department of Health and Human Services). Psychosocial stress was defined as a combined measure of general stress at home and at work (permanent or several periods of stress versus no or some periods of stress in the past year). Depression was defined as feeling sad, blue, or depressed for 2 or more consecutive weeks during the past year.

Nontraditional risk factors that have been increasingly recognized as relevant contributors to ischemic stroke, particularly in the younger population,^13^ included a history of venous thrombosis, malignancy, and any chronic multisystem disorder, such as autoimmune disease, inflammatory bowel disease, chronic kidney disease, chronic liver disease, or hematologic disease/thrombophilia. Furthermore, we recorded the presence of migraine with aura, assessed with a validated standardized questionnaire,^15^ and current use of illicit drugs with the Alcohol, Smoking and Substance Involvement Screening Test questionnaire.^28^

Available female sex–specific risk factors^29^ included a history of perinatal conditions including gestational diabetes, gestational hypertension, pregnancy complications (preeclampsia, eclampsia, or hemolysis, elevated liver enzymes, and low platelet count syndrome), and current pregnancy or puerperium. Furthermore, current estrogen use through any route of administration was recorded.

In addition, we defined behavioral risk factors as additional clinically actionable group of risk factors, which included current smoking, abdominal obesity, physical inactivity, unhealthy diet, heavy alcohol use, illicit drug use, psychosocial stress, and in women, estrogen use.

### Statistical Analysis

Statistical analyses used IBM SPSS Statistics 29.0 and R (R Core Team 2023). *P*<0.05 were considered significant. Missing data handling is described in the Supplemental Methods.

Clinical characteristics in patients with CIS with and without PFO were compared with those of all stroke-free controls. Among patients with CIS only, clinical characteristics were compared between those with and without PFO. Based on their distributions, counts of the 12 traditional risk factors were described in categories of none, 1, 2, 3, and ≥4. Count of the 9 nontraditional risk factors was categorized as none, 1, and ≥2, whereas the presence of any female sex–specific risk factor was described as a binary variable. Risk factor counts were chosen to quantify the overall burden of risk factors in a simple, interpretable manner, and provide a cumulative estimate of the exposure. Pearson χ^2^, Fisher exact, Mann-Whitney *U* test, and Wilcoxon rank-sum tests were used for univariable comparisons. To address potential bias produced by nonstandardized methods of identifying controls, we performed a sensitivity analysis comparing control subjects identified from population-based sources versus nonpopulation-based sources.

In patients with CIS with and without PFO, our primary multivariable analyses consisted of risk factor counts in logistic regression models rather than conditional regression models, as maintaining 1-to-1 matching between cases and controls was not applicable. The linearity assumption for risk factor counts was assessed using logits and confirmed. Models were adjusted for age, sex, and level of education. The first model included the count of traditional risk factors, whereas the second model incorporated counts of both traditional and nontraditional risk factors. For women, a third model was constructed by adding female sex–specific risk factors. To evaluate demographic differences in the contribution of these risk factors, separate models were fitted for women, men, and 2 predefined age groups (18–39 and 40–49 years). In addition, interactions between risk factor counts and both sex and categorical age were tested to assess potential effect modification.

We secondarily examined associations of individual risk factors using models adjusted for age, sex, level of education, traditional risk factors, and nontraditional risk factors. These models were applied separately to women, men, and the predefined age groups. In women, the model also incorporated female sex–specific risk factors.

We calculated population-attributable risks (PAR) for both risk factor counts and individual risk factors using the Bruzzi method,^30^ based on logistic regression, and adjusted for confounding factors. CIs for the PARs were estimated using the jackknife method from the R package attribrisk. PARs were derived for traditional, nontraditional, female sex–specific, behavioral, and all combined risk factors.

Among patients with CIS only, we conducted a logistic regression to identify risk factors associated with the phenotype characterized by a higher overall burden of risk factors, using the absence of PFO as the dependent variable. Models examining individual risk factors were adjusted for all other risk factors, while models including risk factor counts were adjusted for counts of traditional and nontraditional risk factors. In females, sex-specific risk factors were also included.

## Results

Of the initially enrolled 546 patients, 23 patients were excluded due to poor visualization or technical difficulties in echocardiography, which prevented the determination of PFO status. Thus, 523 patients with CIS (median age, 41 years; 47.2% women) and 523 age- (±5 years) and sex-matched stroke-free controls (median age, 41 years; 47.2% women) were included in the analysis. Female patients were younger than males (median, 40 versus 42 years; *P*<0.001). A total of 196 (37.5%) patients had PFO, with no difference in its prevalence across sexes and age groups. Results of the sensitivity analysis comparing population-based and nonpopulation-based controls appear in the Supplemental Results.

### Prevalence of Risk Factors

Comparison of clinical characteristics between stroke-free controls and patients with and without PFO is given in Table 1. No differences emerged in age and sex distribution between the groups. Compared with stroke-free controls, patients with CIS without PFO more frequently had a low level of education, and higher prevalence of most traditional risk factors, including cardiovascular disease, hypertension, current smoking, abdominal obesity, physical inactivity, unhealthy diet, heavy alcohol use, psychosocial stress, and depression. Patients with CIS with PFO had a significantly higher prevalence of current smoking and abdominal obesity than did controls. Among the nontraditional risk factors, a history of venous thrombosis and migraine with aura were more frequent in patients without PFO compared with stroke-free controls. Patients with PFO also had a higher prevalence of migraine with aura compared with stroke-free controls. Among patients with CIS alone, those without PFO more frequently had a low level of education, obstructive sleep apnea, hypertension, an unhealthy diet, current smoking, and heavy alcohol use, as compared with CIS with PFO. Among the nontraditional risk factors, migraine with aura was more common in patients with CIS with PFO.

**Table 1.**
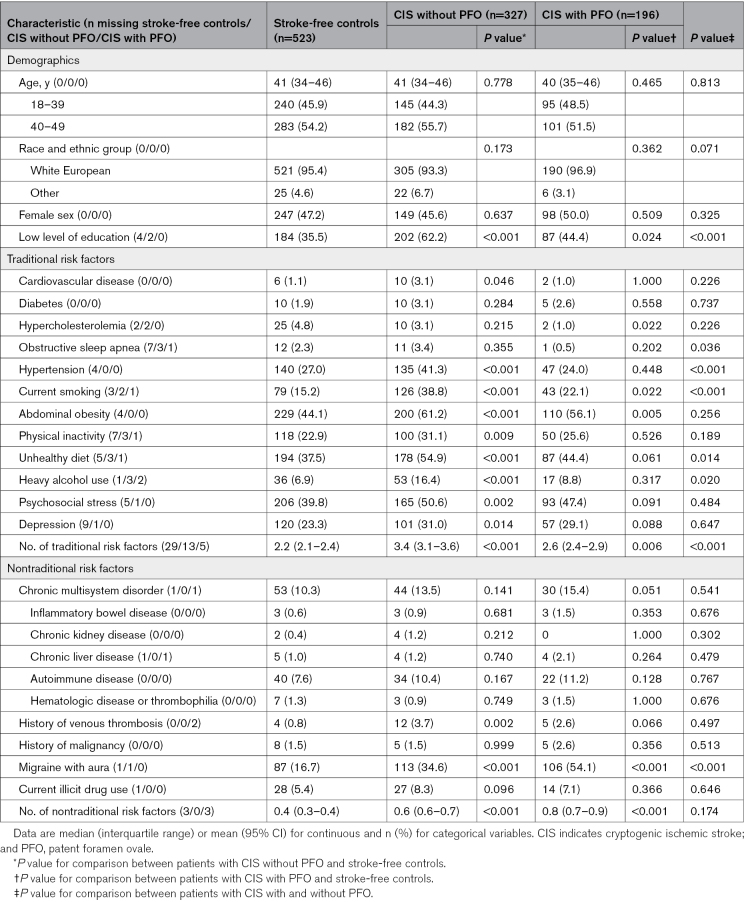
Demographic Characteristics and Risk Factors of Patients With CIS Stratified by the Presence of High-Risk PFO, With Comparison of Patients With CIS to Stroke-Free Healthy Controls and Patients With CIS With and Without PFO

**Figure 1.**
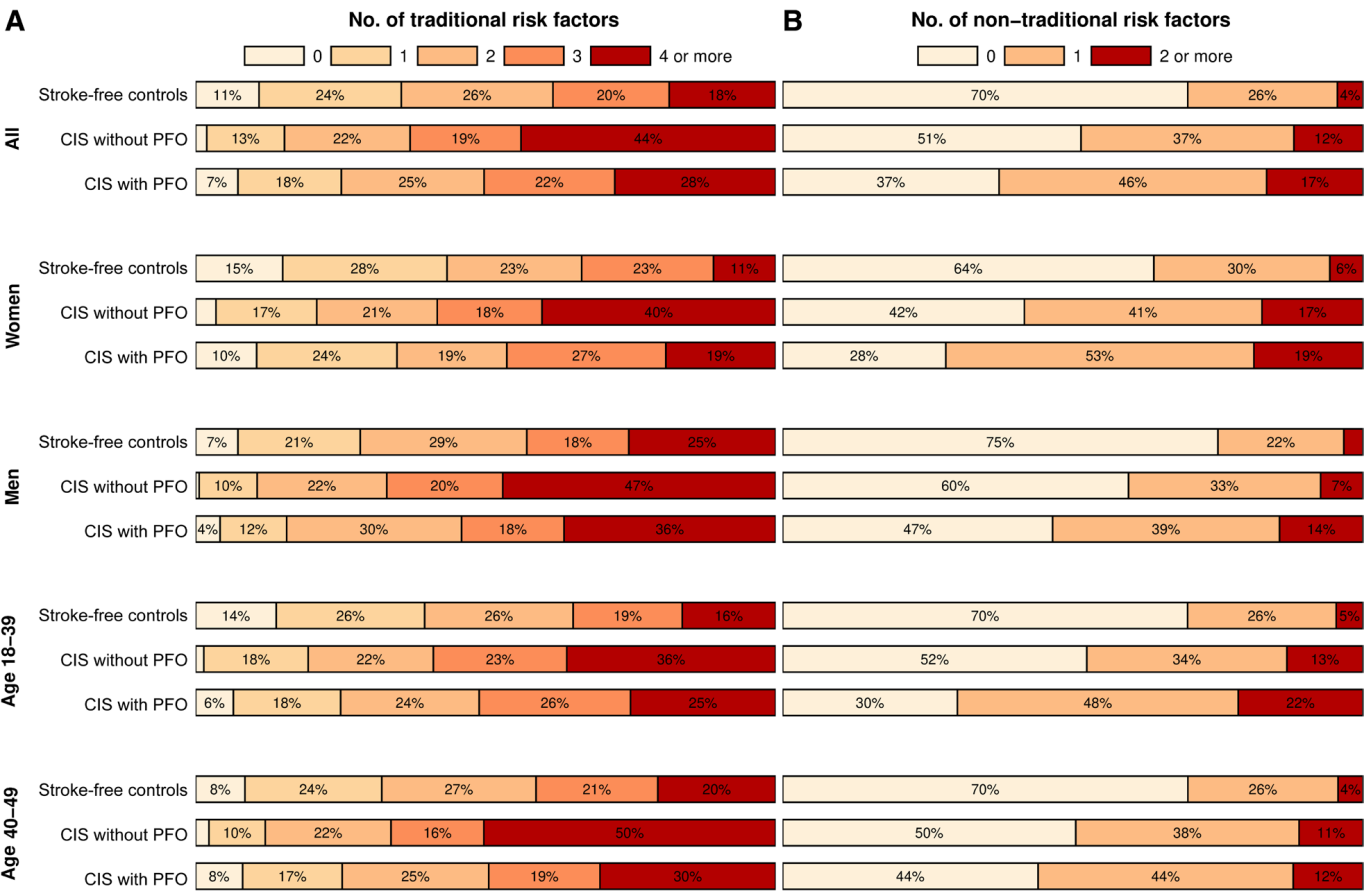
**Proportions of traditional and nontraditional risk factor counts in the overall cohort, women, men, and age groups. A**, Traditional risk factors; (**B**) nontraditional risk factors. Results presented for stroke-free controls and patients with cryptogenic ischemic stroke (CIS) stratified by the presence of high-risk patent foramen ovale (PFO).

When compared with stroke-free controls, both patients with CIS with and without PFO had higher counts of traditional and nontraditional risk factors. The difference in the count of traditional risk factors was more profound in patients with CIS without PFO and the count of nontraditional risk factors was more profound in patients with CIS with PFO, compared with controls. Patients with CIS without PFO had a higher count of traditional risk factors than patients with CIS with PFO. The count of nontraditional risk factors did not differ significantly by the presence of PFO (Figure 1). Demographics and frequencies of individual risk factors for patients with CIS with and without PFO and stroke-free controls by sex are presented in Table S1 (women) and Table S2 (men), as detailed in the Supplemental Results.

Across both sexes, counts of traditional risk factors were higher in patients with CIS with and without PFO compared with stroke-free controls. In addition, female patients with CIS with and without PFO showed a higher count of female sex–specific risk factors compared with female stroke-free controls. Among both female and male patients with CIS, the count of traditional risk factors was higher in those without PFO than in patients with PFO. The count of nontraditional risk factors was significantly higher only in male patients with PFO compared with those without PFO (Figure 1). Demographics and frequencies of individual risk factors for patients with CIS with and without PFO and stroke-free controls by age group are presented in Table S3 (ages, 18–39 years) and Table S4 (ages, 40–49 years), with further details in the Supplemental Results.

Across both age groups, counts of traditional risk factors were higher in patients with CIS without PFO compared with stroke-free controls. Compared with stroke-free controls, counts of nontraditional risk factors were higher in patients with CIS with PFO aged 18 to 39 years but not among those aged 40 to 49 years. Among patients with CIS aged 18 to 39 years, the count of traditional risk factors did not differ significantly between those with and without PFO, whereas the count of nontraditional risk factors was higher in patients with CIS with PFO. Among patients with CIS aged 40 to 49 years, the opposite was observed (Figure 1).

### Counts of Risk Factors

**Figure 2.**
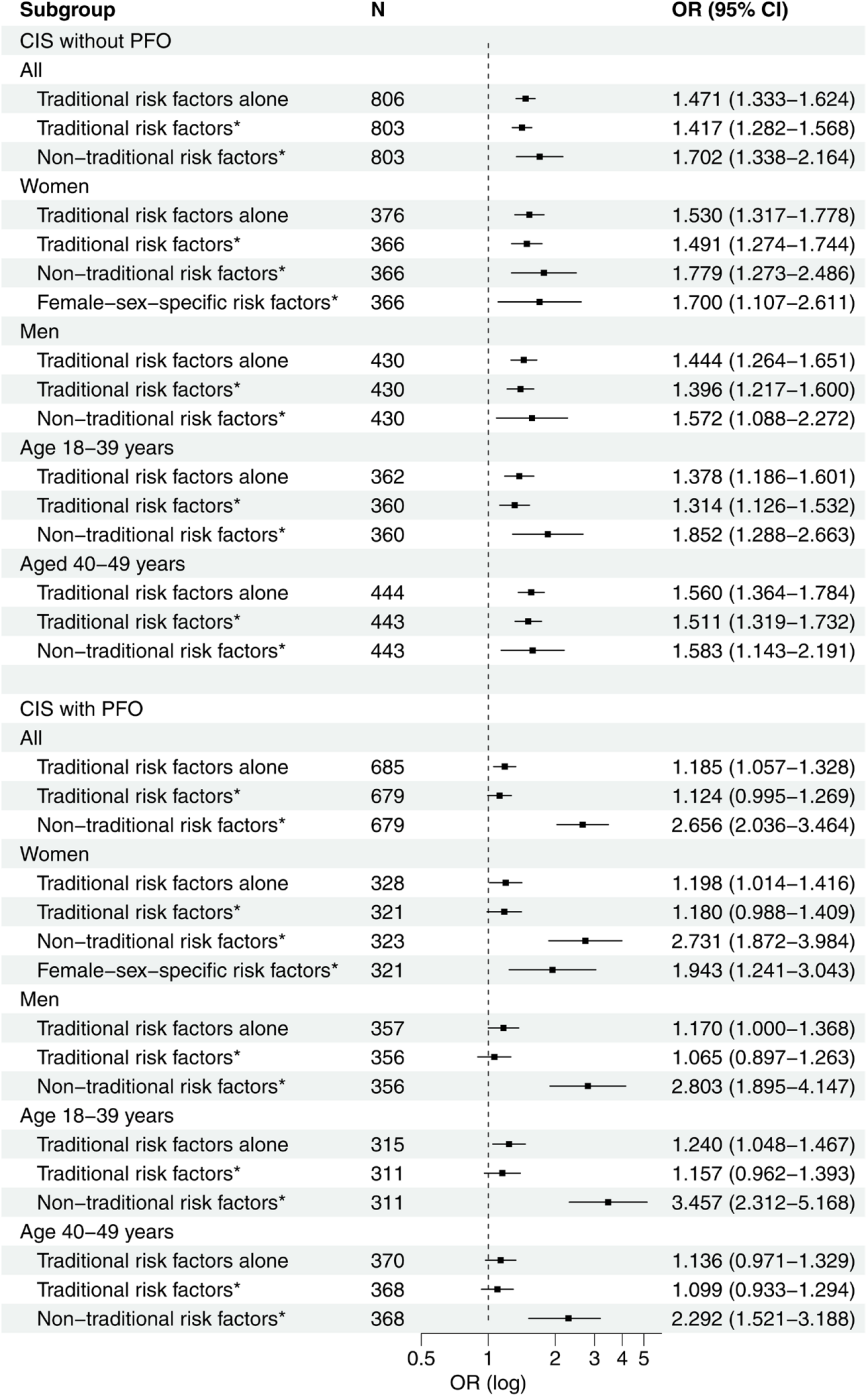
**Adjusted odds ratios (OR) with 95% CIs for the association of each incremental traditional, nontraditional, and female sex–specific risk factor with cryptogenic ischemic stroke (CIS) in the entire cohort and by sex and age group, stratified by the presence of high-risk patent foramen ovale (PFO) in patients.** All models were adjusted for demographics and other risk factor count(s), except the models including traditional risk factors alone. *Fully adjusted models.

When assessing risk factors in patients with CIS without PFO, adjusted logistic regression models showed associations for each incremental traditional (odds ratio, 1.4 [95% CI, 1.3–1.6]) and nontraditional (odds ratio, 1.7 [95% CI, 1.3–2.2]) risk factor, with consistent point estimates and overlapping CIs across sexes and age groups. There was no notable attenuation of the strength of association of traditional risk factor count after adjustment for nontraditional risk factor count, and in women, count of female sex–specific risk factors. Among women, the strength of association of female sex–specific risk factors was of the same order as for traditional and nontraditional risk factors (Figure 2; see Table S5 for unadjusted estimates). We found no formal interactions between age or sex and count of traditional risk factors (*P* for interaction=0.248 and *P* for interaction=0.464, respectively), and no interactions between age or sex and count of nontraditional risk factors (*P*=0.680 and *P*=0.685, respectively).

Regarding risk factors in patients with CIS with PFO, the association of the count of traditional risk factors was significant only in models adjusted for demographics in the overall cohort, across sexes, and in the younger age group, while further adjustment attenuated this association. In contrast, nontraditional risk factors showed consistently strong associations in the overall cohort (odds ratio, 2.7 [95% CI, 2.0–3.5]) and across demographic subgroups. Furthermore, each additional female sex–specific risk factor increased the odds of CIS with PFO by 1.9 (95% CI, 1.2–3.0; Figure 2). We found no interactions between age or sex and count of traditional risk factors (*P* for interaction=0.805 and *P* for interaction=0.541, respectively), and no interactions between age or sex and count of nontraditional risk factors (*P*=0.069 and *P*=0.991, respectively).

### Contribution of Risk Factors

**Figure 3.**
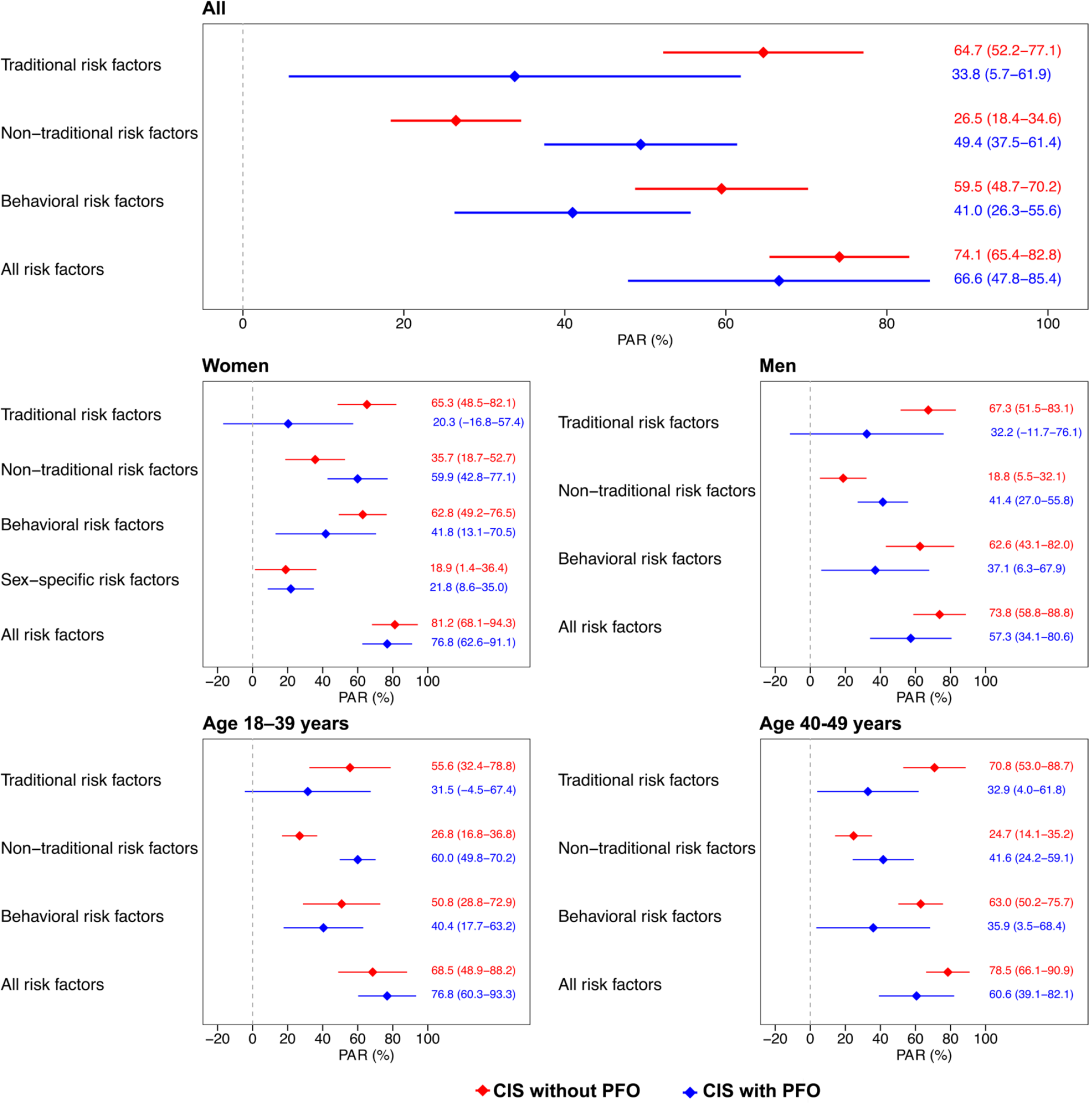
**Adjusted population-attributable risks (PARs) and corresponding 95% CIs for combinations of risk factors in the entire cohort and by sex and age group, stratified by the presence of high-risk patent foramen ovale (PFO) in patients with cryptogenic ischemic stroke (CIS).** Logistic regression models used to generate PARs were adjusted for age, sex (when appropriate), level of education, and other risk factor counts.

In the entire cohort, nontraditional risk factors showed a stronger contribution to CIS among patients with PFO compared with those without PFO (PAR 49.4% versus 26.5%). PAR point estimates of traditional risk factors suggested a stronger contribution to CIS among patients without PFO than those with PFO (PAR 64.7% versus 33.8%, respectively), although with overlapping confidence limits. The magnitudes of sex-specific PARs were in line with those of the entire cohort, with slightly lower PARs for nontraditional risk factors in men than in women. Female sex–specific risk factors contributed equally to the risk for CIS in patients with and without PFO (PAR 18.9% and 21.8%, respectively). Among those aged 18 to 39 years, the difference in contribution of nontraditional risk factors in the development of CIS was more profound in patients with than without PFO in this age group compared with those aged 40 to 49 years (Figure 3).

The PAR point estimates for the combination of behavioral risk factors suggested a stronger contribution to CIS in patients with PFO compared with those without (59.5% versus 41.0%, respectively). Notably, the contributions of traditional and behavioral risk factors were similar overall and within each demographic subgroup in the PFO strata (Figure 3).

**Figure 4.**
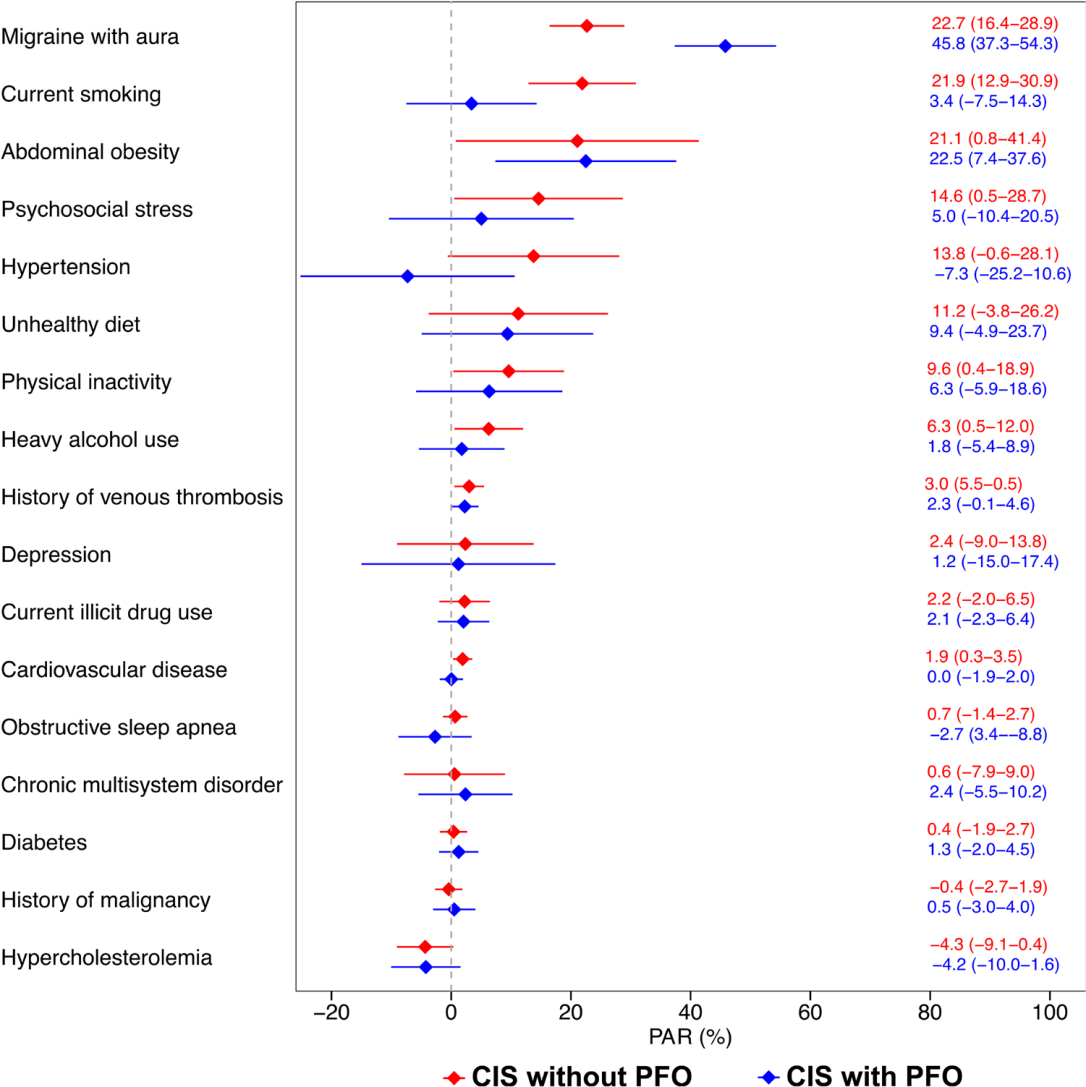
**Adjusted population-attributable risks (PARs) with 95% CIs for individual risk factors in the overall cohort, stratified by the presence of high-risk patent foramen ovale (PFO) in patients with cryptogenic ischemic stroke (CIS).** Logistic regression models used to generate PARs were adjusted for age, sex (when appropriate), level of education, and all risk factors.

Associations of individual risk factors are presented in Table S6 and described in the Supplemental Results. In terms of PARs, the top 3 individual traditional risk factors contributing to the development of CIS among patients without PFO were migraine with aura (22.7%), current smoking (21.9%), and abdominal obesity (21.1%). The leading risk factors contributing to CIS among patients with PFO were migraine with aura (PAR, 45.8%), abdominal obesity (PAR, 22.5%), and unhealthy diet (PAR, 9.4%; Figure 4).

Associations of individual risk factors by sex are presented in Tables S7 and S8, as described in the Supplemental Results. The top contributors in female patients were migraine with aura (PAR 29.3% for patients with CIS without PFO versus 55.5% for patients with CIS with PFO), unhealthy diet (22.0% versus 7.4%), and abdominal obesity (20.3% versus 19.4%). In male patients, the highest PARs appeared for current smoking (29.3% versus 2.0%), abdominal obesity (21.3% versus 16.3%), and psychosocial stress (19.8% versus 17.7%) for patients without and with PFO, respectively. Notably, migraine with aura showed a lower PAR for male patients without PFO than with PFO (17.7% versus 37.2%; Figure S1).

Associations of individual risk factors by age group are presented in Tables S9 and S10, as described in the Supplemental Results. In the younger age group, abdominal obesity (PAR 26.0% or patients without PFO and 28.8% for patients with PFO), migraine with aura (19.4% versus 52.7%), and chronic multisystem disorder (9.7% versus 10.1%) contributed most to the CIS risk. In the older age group, the top risk factors were migraine with aura (PAR 26.7% for patients without PFO versus 40.6% for patients with PFO), current smoking (25.0% versus 7.9%), and hypertension (24.1% versus −5.1%; Figure S2).

### Factors Associated With the Absence of PFO Among Patients With CIS

Among patients with CIS, independent factors associated with the absence of PFO in the logistic regression were hypertension and current smoking, and the absence of migraine with aura. Each additional traditional risk factor was associated with ≈30% higher probability of not having a PFO, whereas each additional nontraditional risk factor was associated with 33% lower probability of not having a PFO. Among female patients, only the absence of migraine was independently associated with not having a PFO. Among male patients, hypertension, current smoking, and the absence of migraine and depression were associated with the absence of PFO. Hypertension was associated with the absence of PFO across both age groups, while migraine with aura was associated with the absence of PFO in the younger age group (Table 2; unadjusted results in Tables S11 through S13).

**Table 2.**
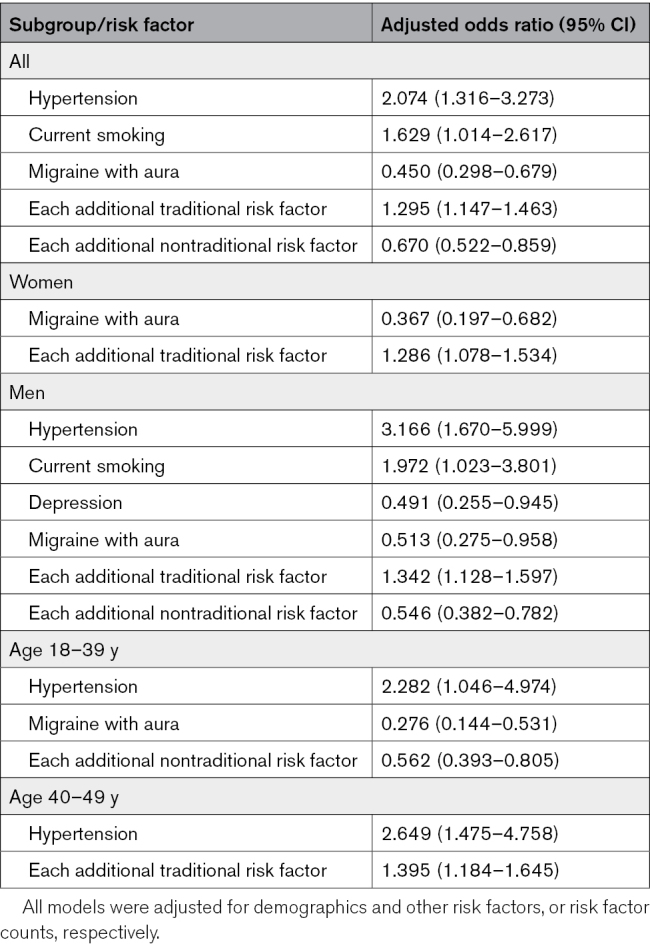
Results From Adjusted Logistic Regression Analyses on the Independent Individual Risk Factors and Risk Factor Counts Showing Significant Associations With Cryptogenic Ischemic Stroke Without High-Risk Patent Foramen Ovale

## Discussion

In this study of young adults with CIS, we found that both traditional and nontraditional risk factors contribute significantly to the development of CIS, with notable differences depending on the presence of a clinically relevant PFO defined as those with high-risk features. Traditional risk factors were strongly associated with CIS without PFO, whereas nontraditional risk factors played a more prominent role among patients with CIS with PFO. Migraine with aura emerged as the most prominent individual risk factor for CIS, among patients with and without PFO, particularly in female patients.

Only a few case-control studies have assessed the impact of risk factor burden on young-onset ischemic stroke. A German study comparing 2125 young stroke cases (94% ischemic strokes) to 8500 controls showed that traditional cardiovascular risk factors are significant contributors. Low physical activity and hypertension were the most important risk factors for ischemic stroke, accounting for 59.8% and 25.2% of the risk, respectively, with behavioral risk factors combined explaining 71.0% of the risk.^8^ In our study, behavioral risk factors contributed to the risk of CIS to the same extent as all traditional risk factors combined, but notably, behavioral risk factors combine both individual traditional and nontraditional risk factors. A US study found that stroke risk increased exponentially with the number of concurrent vascular risk factors: odds ratios were 2.1 for 1 risk factor, 2.6 for 2, 7.6 for 3, and 16.5 for 4, with Black patients ≈6× more likely to have all 4 major risk factors compared with White patients.^14^ Another study demonstrated that atherosclerotic risk factors and atherosclerosis become more impactful with age but were already important at younger ages, affecting 8.5% of ischemic stroke cases in ages 20 to 29 years and rising to 42.5% in ages 40 to 49 years.^10^ A recent US study based on administrative claims explored both traditional and nontraditional risk factors, finding that nontraditional factors, such as migraine, play a significant role in strokes among younger adults, especially female survivors. The influence of nontraditional factors declined with age, while traditional factors became more prominent.^13^ However, the former studies did not include nontraditional risk factors, and the latter did not have accurate ascertainment of risk factors because it was performed on an administrative data set. This study assessed traditional, nontraditional, and female sex–specific risk factors and focused specifically on their association with young-onset CIS. Our study aligns with previous research demonstrating the cumulative impact of risk factor burden on the risk of ischemic stroke, extending this evidence specifically to the main clinical young-onset CIS subtypes, both with and without PFO.

Among young adult patients with ischemic stroke the prevalence of PFO is known to vary according to the prevalence of traditional risk factors,^31^ One study specifically assessing the number of traditional atherosclerosis risk factors in young-onset CIS suggested that the influence of the interatrial right-to-left shunt (primarily PFO) on CIS risk diminishes as the number of traditional risk factors increases, indicating a potential inverse association.^19^ However, this and other studies have used varying definitions of PFO, often considering any grade of right-to-left shunt as PFO. This broad definition of PFO does not align with the more clinically relevant concept of PFO defined as having high-risk features which we used in our study. PFO with high-risk features is clinically relevant because of its association with a higher likelihood of causing a stroke, leading to the decision on whether to recommend PFO closure.^20^ Nevertheless, our findings reinforce the idea that traditional and nontraditional risk factors play distinct roles in young-onset patients with CIS, depending on the presence of a clinically relevant PFO.

Earlier data have indicated that young patients with CIS with PFO have a higher prevalence of migraine, further suggesting different stroke mechanisms in those with and without PFO.^18^ A recent case-control study by Leppert et al^13^ emphasized migraine as the most important nontraditional risk factor for young-onset stroke, with a stronger impact in younger individuals and female survivors, surpassing traditional risk factors, such as hypertension and tobacco smoking. However, this study was limited by the lack of specific clinical criteria to define migraine and could not distinguish between all types of migraine or migraine with aura, the latter of which is more strongly associated with ischemic strokes. Although it remains unclear whether cryptogenic cases are primarily responsible for this association, our findings align with and expand upon these observations, identifying migraine with aura as the leading individual risk factor for young-onset CIS. This risk is significant regardless of the presence of PFO but is particularly pronounced in patients with CIS with PFO, women, and younger patients. The potential mechanisms linking migraine and CIS have been extensively discussed,^13,32^ and our findings, alongside others, strongly advocate for further research to unravel the pathogenesis underlying the increased stroke risk in individuals with migraine. Furthermore, interactions between migraine with aura and other individual risk factors merit further investigation.

To our knowledge, this study is the first to provide sex- and age-specific estimates on the contribution of a broad range of traditional, nontraditional, and female-specific risk factors to young-onset CIS, stratified by the presence of clinically relevant PFO. Notably, the extent to which these risk factors explain young-onset CIS is comparable to, and even close to the upper range for, young-onset ischemic stroke of any pathogenesis.^8,13^ This challenges the historical view that cryptogenic cerebrovascular events have limited explanations. Importantly, some traditional risk factors (eg, psychosocial stress, unhealthy diet, heavy drinking) are often underrepresented in medical records or lack accuracy, requiring extra effort to capture them. Our findings underscore the need to systematically screen not only traditional risk factors with sufficient detail but also nontraditional and female-specific factors, which often receive too little attention. This comprehensive approach is crucial for planning preventive strategies for young individuals, even those who appear to have low stroke risk. In addition, identifying clinically relevant PFO can help target interventions, such as more aggressive management of traditional risk factors in young patients with CIS without PFO. Nevertheless, it should be noted that some of the individual risk factor associations observed in our study were only weak or moderate, and not all risk factors, such as migraine with aura, are convincingly modifiable.

Our study has several strengths, including its large, well-defined cohort of young adults with CIS enrolled in a multicenter study, inclusion of consecutive patients irrespective of PFO status, and comprehensive assessment of traditional and nontraditional risk factors, including those specific to women. Stratification by clinically relevant PFO provides valuable insights into the differential impact of risk factors in young-onset CIS. In addition, the inclusion of behavioral risk factors, often underreported in prior research, adds depth to the analysis. However, the study also has limitations. The cross-sectional design precludes causal inferences, and reliance on self-reported data for behavioral risk factors may introduce recall bias. Although we stratified by PFO status, the absence of further anatomic high-risk PFO features and related clinical factors may limit classification accuracy. Stroke-free controls were not stratified by PFO status and while their lack of knowledge about PFO status at the time of assessment minimized bias, certain risk factors—such as migraine with aura—are associated with PFO. Given that stroke-free controls likely have fewer high-risk PFOs, some associations between CIS and risk factors may partially reflect PFO itself rather than stroke risk alone. Selection bias cannot be excluded due to variability in control recruitment methods, although risk factor frequencies in controls closely mirror those in the general population. Sensitivity analysis revealed minor differences in risk factor prevalence between population-based and nonpopulation-based controls, but these were unlikely to affect conclusions. Multiple univariable comparisons were used to descriptively characterize baseline differences, without corrections for multiple comparisons, as they were not the basis of our conclusions. Primary findings rely on multivariable analyses, which adjust for confounding factors and provide robust estimates. Lack of power limited the detection of associations for rare risk factors and smaller subgroups. We underestimated the prevalence of certain conditions, such as hyperlipidemia, diabetes, obstructive sleep apnea, and prothrombotic disorders, as their definitions relied only on history. Finally, although the study’s focus on young adults is a strength, the findings may not be generalizable to older populations or those with different racial, ethnic, or geographic backgrounds. Notably, 95% of our participants were White Europeans, and although this reflects the demographic composition of the study sites, it limits the applicability of our results to more diverse populations. Stroke risk factors may vary across racial and ethnic groups due to genetic, environmental, and socioeconomic factors.

## Conclusions

Our study highlights the multifaceted nature of young-onset CIS, revealing that both traditional and nontraditional risk factors significantly influence its development, with variations based on clinically relevant PFO status. The prominent role of behavioral factors and the strong association with migraine with aura underscore the necessity for a thorough and tailored approach to risk factor assessment and prevention strategies in young adults. However, to gain a deeper understanding of the complex physiological and molecular mechanisms involved, including the interplay between migraine and other risk factors, and how these may differ by PFO status, further studies are warranted.

## ARTICLE INFORMATION

### Acknowledgments

The authors are indebted to Anu Eräkanto for substantial technical help, study nurses, and all study participants for their volunteer participation in the study.

### Sources of Funding

Supported by Helsinki and Uusimaa Hospital District research fund (TYH2014407 and TYH2018318); Academy of Finland (286246, 318075, and 322656); Sahlgrenska University Hospital (ALFGBG-726821), Finnish Medical Foundation, and Sigrid Juselius Foundation.

### Disclosures

None.

### Supplemental Material

Tables S1–S14

Figures S1–S2

STROBE Checklist

## APPENDIX

SECRETO Study Group (Principal Investigator [PI])

Tartu University Hospital, Tartu, Estonia: Külliki Karu, Janika Kõrv (PI), Liisa Kõrv, Piibe Muda, Riina Vibo; Helsinki University Hospital, Helsinki, Finland: Mika Lehto, Nicolas Martinez-Majander, Jani Pirinen, Jukka Putaala (PI), Janne Rapola, Tiina Sairanen, Juha Sinisalo, Lauri Soinne, Satu Suihko, Marjaana Tiainen, Heli Tolppanen, Lauri Tulkki, Suvi Tuohinen; Kuopio University Hospital and University of Eastern Finland, Kuopio, Finland: Jaana Autere, Marja Hedman, Nina Jaakonmäki, Pekka Jäkälä (PI), Tuuli Miettinen, Ossi Nerg; Tampere University Hospital, Tampere, Finland: Heikki Numminen, Essi Ryödi, Tomi Sarkanen (PI), Marko Virtanen; Turku University Hospital and University of Turku, Turku, Finland: Riitta Lautamäki, Antti Saraste, Pauli Ylikotila (PI); Oulu University Hospital, Oulu, Finland: Jaana Huhtakangas, Juha Huhtakangas (PI), Ulla Junttola, Laura Kytövuori; University Medical Greifswald, Greifswald, Germany: Raila Busch, Ulf Schminke, Bettina von Sarnowski (PI); Attikon University Hospital, Athens, Greece: Alexandra Frogoudaki, Georgios Papadimitripoulos, Georgios Tsivgoulis (PI); Azienda Unità Sanitaria Locale–IRCCS, Reggio Emilia, Italy: Teresa Grimaldi, Giovanni Malferrari, Marialuisa Zedde (PI); University of Brescia, Brescia, Italy: Marina Colombi, CarloMario Lombardi, Alessandro Pezzini (PI), Marco Ritelli; Vilnius University Hospital, Vilnius, Lithuania: Aleksandra Ekkert, Dalius Jatuzis, Rytis Masiliunas, Kristina Ryliskiene (PI), Diana Zakarkaite; Radboud University Medical Center, Nijmegen, the Netherlands: Frank-Erik de Leeuw (PI), Merel Ekker, Suzette Elias-Smale, Myrna M.E. van Dongen; Haukeland University Hospital, Bergen, Norway: Annette Fromm, Eva Gerdts, Sahrai Saeed, Ulrike Waje-Andreassen (PI); Hospital de Santa Maria, University of Lisbon, Lisbon, Portugal: Ana G. Almeida, Isabel Amorim, José Manuel Ferro, AnaCatarina Fonseca (PI), André Paula; Torrecardenas University Hospital, Spain: Patricia Martínez-Sánchez (PI), Victoria Mejias Olmedo, Laura Amaya Pascasio, Raul Reyes Parrilla, Elvira Carrión Rios; Sahlgrenska Academy at University of Gothenburg and Sahlgrenska University Hospital, Gothenburg, Sweden: Margareta Abrahamson, Odd Bech-Hanssen, Maria Davidson, Lukas Holmegaard, Mikael Jerndal, Katarina Jood, Annika Nordanstig, Petra Redfors (PI), Turgut Tatlisumak; Istanbul University, Istanbul, Turkey: Esme Ekizoglu Turgut, Sezgin Mine, Nilufer Yesilot (PI); Royal Stoke University Hospital, Stoke-on-Trent, United Kingdom: Phillip Ferdinand (PI), Cheryl Oxley, Zoltan Pencz, Christine Roffe; Peterborough City Hospital, Peterborough, United Kingdom: Mohammad Hacque, Muhammad Khaled Hasan, Radim Licenik (PI), Peter Owusu-Agyei, Santhosh Subramonian
